# Validating Small-Molecule Force Fields for Macrocyclic Compounds Using NMR Data in Different Solvents

**DOI:** 10.1021/acs.jcim.4c01120

**Published:** 2024-10-15

**Authors:** Franz Waibl, Fabio Casagrande, Fabian Dey, Sereina Riniker

**Affiliations:** † Department of Chemistry and Applied Biosciences, ETH Zürich, Vladimir-Prelog-Weg 2, 8093 Zürich, Switzerland; ‡ Roche Pharma Research and Early Development, Therapeutic Modalities, Roche Innovation Center Basel, F. Hoffmann-La Roche, 4070 Basel, Switzerland

## Abstract

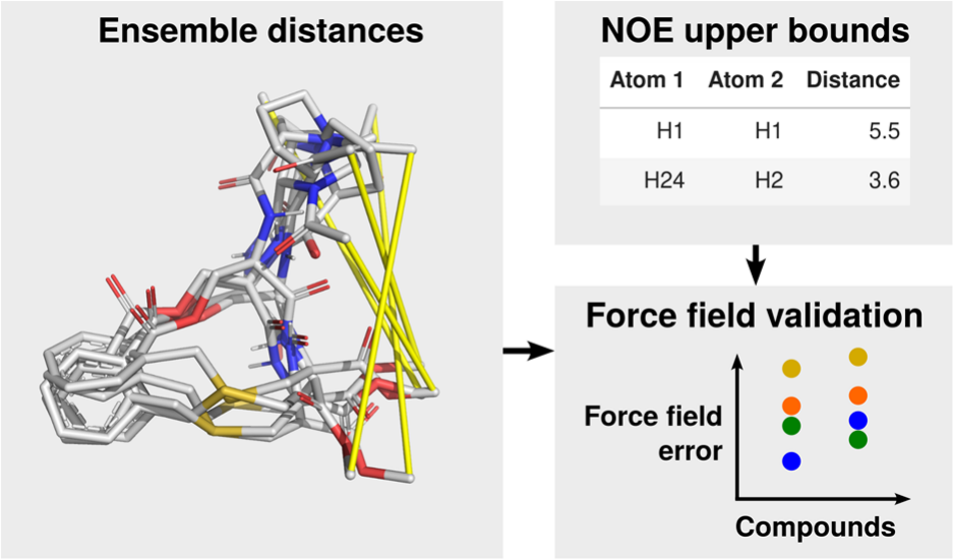

Macrocycles are a promising class of compounds as therapeutics for difficult drug targets due to a favorable combination of properties: They often exhibit improved binding affinity compared to their linear counterparts due to their reduced conformational flexibility, while still being able to adapt to environments of different polarity. To assist in the rational design of macrocyclic drugs, there is need for computational methods that can accurately predict conformational ensembles of macrocycles in different environments. Molecular dynamics (MD) simulations remain one of the most accurate methods to predict ensembles quantitatively, although the accuracy is governed by the underlying force field. In this work, we benchmark four different force fields for their application to macrocycles by performing replica exchange with solute tempering (REST2) simulations of 11 macrocyclic compounds and comparing the obtained conformational ensembles to nuclear Overhauser effect (NOE) upper distance bounds from NMR experiments. Especially, the modern force fields OpenFF 2.0 and XFF yield good results, outperforming force fields like GAFF2 and OPLS/AA. We conclude that REST2 in combination with modern force fields can often produce accurate ensembles of macrocyclic compounds. However, we also highlight examples for which all examined force fields fail to produce ensembles that fulfill the experimental constraints.

## Introduction

The properties of molecules in solution are of great interest for the investigation of biological systems or the development of new therapeutics. Pharmacokinetic properties such as aqueous solubility or passive membrane permeability are important for the latter,^1^ with guidelines such as Lipinski’s rule of five (Ro5)^2^ or Veber’s rule^3^ presenting recommendations for the rational design of orally available drugs. However, these rules can often not be fulfilled by larger compounds. While newer drugs increasingly exceed the Ro5 limits (especially in terms of molecular weight^4−6^), it is still challenging to obtain drug-like properties and especially oral bioavailability with larger compounds.^7^ It has been observed that macrocyclic compounds (i.e., compounds with a ring of 12 or more atoms) can exhibit a favorable combination of passive membrane permeability,^8,9^ binding affinity, and solubility^10,11^ compared to their acyclic counterparts of similar size and composition.^12^ This extends their drug-like range^13^ and makes them promising candidates for the development of novel therapeutics. Although several *de novo* designed scaffolds have been reported^14^ using computational tools to predict and optimize the membrane permeability of the novel compounds,^15,16^ most macrocyclic drugs so far are derived from natural products,^17^ in part because of the difficulty of predicting the binding affinity and pharmacokinetic properties of novel compounds.^18^

The ability of macrocycles to adapt their apparent polarity to that of the surroundings by conformational change^19^ has been termed chameleonicity,^20,21^ with cyclosporine A being the most thoroughly investigated example.^22,25^ In an apolar environment (such as the interior of a cell membrane), the polar groups are shielded from the surroundings through intramolecular hydrogen bonds. Chameleonicity significantly increases the passive membrane permeability of a compound in the presence of a relatively large number of hydrogen-bond donors and acceptors, which are required for good solubility and target binding.^10^ Note that chameleonicity is not limited to macrocycles, it can occur in any sufficiently flexible molecule and several nonmacrocyclic examples exist.^20^

Since the conformational behavior of such compounds is inherently dynamic and depends on the environment, reliable physical models are needed to enable in silico predictions of their properties via conformational ensembles.^23^ Molecular dynamics (MD) simulation is a well-established method to obtain structural ensembles of molecular systems,^24^ and has already been applied to study the conformations of (mostly peptidic) macrocycles.^25−28^ The accuracy of the generated ensembles depends thereby crucially on the accuracy of the underlying force field.^29^

In this study, we assess the performance of MD simulations at generating accurate conformational ensembles of nonpeptidic and semipeptidic macrocyclic compounds by comparing the results with nuclear Overhauser effect (NOE) upper distance bounds from NMR experiments in chloroform, water, or DMSO. We compare between four popular force fields for organic molecules: the second version of the general AMBER force field^30^ (GAFF2), OPLS/AA,^31^ OpenFF version 2.0.0 (Sage),^32^ and the recently reported XFF force field^33^ in combination with DASH^34^ partial charges, on 11 public and six in-house compounds. To ensure sufficient sampling, we employ the sampling-enhancement technique replica exchange with solute scaling (REST2),^35^ which is based on replica-exchange MD (REMD).^36^ We show that REST2 can be used to sample the conformational space of macrocycles. In several cases, we find excellent agreement with the experimental data, while also highlighting cases where the ensembles violate the experimental constraints. Additionally, we compare two different schemes to distribute replicas in REST2, showing that the quadratic scheme performs better. We also find that bond-angle terms need to be included in REST2 to ensure proper sampling for one compound with a strained ring system.

## Methods

### Compounds and Reference Data

**Figure 1:**
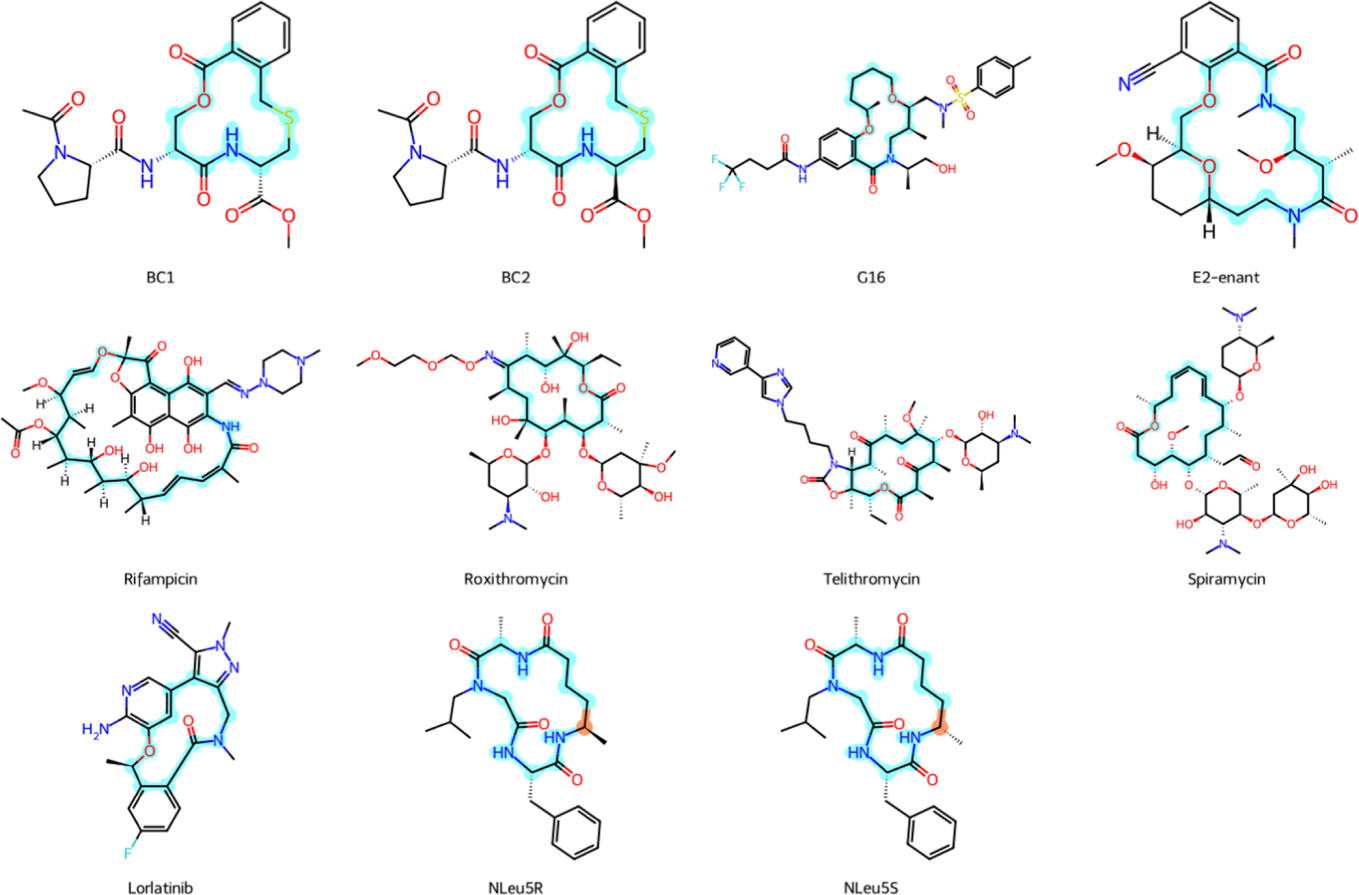
2D depictions of the 11 public molecules used in this study. In each molecule, the macrocycle is highlighted in light blue for clarity, and the stereocenter that distinguishes NLeu5S from NLeu5R is highlighted in orange.

We investigated 11 nonpeptidic or semipeptidic macrocycles for which experimental NOE data is available from the literature (Figure 1): Compounds 1 and 2 from Begnini et al.^37^ (called B1 and B2 in this work), G16 and E2-enant from Poongavanam et al.,^38^ as well as rifampicin, spiramycin, telithromycin, and roxithromycin from Danelius et al.^39^ Furthermore, we used the data for lorlatinib from Peng et al.,^40^ as well as the compounds NLeu5R and NLeu5S from Comeau et al.^41^ NOE data was taken from the respective original publications. Some distance bounds were discarded or renormalized to account for errors, after discussion with the original authors. The details are provided in the Supporting Information.

### Protonation States

For simulations in chloroform and DMSO, all protonatable groups were assigned the neutral charge state. For the five compounds that were simulated in water, the most probable protonation state at pH 7 was used. This means that the tertiary amines in spiramycin, rifampicin, roxithromycin, and telithromycin were set to the protonated state, while E2-enant was kept in a neutral charge state. For rifampicin in water, we also deprotonated the aromatic core according to https://pubchem.ncbi.nlm.nih.gov/compound/Rifampicin-zwitterion, leading to a zwitterionic structure.

Additionally, we tested the OpenFF 2 and GAFF2 force fields on six in-house compounds for validation. While we cannot disclose the full structure of these molecules, Table 1 lists some of their properties.

**Table 1:** Properties of the Six In-House Compounds^a^

name	heavy atoms	H-acceptors	H-donors	ring size	*N*_rot,MC_	*N*_phenyl,MC_
RO1	33	8	5	15	12	0
RO2	37	11	7	17	11	1
RO3	45	10	9	18	14	1
RO4	71	19	13	29	19	2
RO5	71	19	13	32	20	3
RO6	76	22	16	30	19	2

a Abbreviations: *N*_rot,MC_: number of rotatable bonds in the macrocycle; *N*_phenyl,MC_: number of phenyl rings in the macrocycle.

### Conformer Generation

To mimic a scenario where the NMR ensemble is unknown, initial conformers were created using ETKDG version 3^42^ as implemented in the RDKit.^43^ For spiramycin, this simple procedure did not yield a conformer with the correct conformation of the conjugated double bonds. Therefore, we instead generated 500 conformers using ETKDG version 3, and selected the one with the lowest energy after energy optimization with the MMFF94 force field^44,45^ as implemented in the RDKit.

### Force-Field Parameters

Parameters for GAFF2 were assigned using the programs *antechamber* and *tleap* from the AmberTools package version 2022.^46^ AM1-BCC partial charges^47^ were assigned using *antechamber*. Although the default charge model for Amber-type force fields is the restrained electrostatic potential (RESP),^48^ it has been shown that GAFF is compatible both with RESP and AM1-BCC charges.^30^ Here, we decided to use AM1-BCC charges to avoid the expensive Hartree–Fock calculation in RESP, and because of their slightly lower conformer dependence.

OPLS/AA parameters were assigned using the LigParGen web server.^31,49,50^ CM1A charges^49^ were used and three rounds of optimization were performed. Rifampicin could not be parametrized in the neutral state and was therefore omitted in the MD simulations in water with OPLS/AA. For lorlatinib, the dihedral-angle term for the cyano group N≡CC–X had to be removed because it is poorly defined as the cyano group is linear.

OpenFF version 2.0.0 (Sage)^32^ parameters were assigned using the Python interface of the OpenFF toolkit version 0.11.2,^51^ and converted to the GROMACS topology format using OpenFF Interchange version 0.2.2.^52^ AM1-BCC partial charges^47^ were assigned using the OpenFF toolkit with default settings.

XFF parameters were assigned using the XFF web interface at https://xff.xtalpi.com.^33^ Partial charges were assigned using DASH.^34^

For water, TIP3P parameters^53^ were used in all cases. Although it has been shown that newer water models such as OPC^54^ are better at reproducing bulk water properties, TIP3P remains a typical choice for MD simulations, especially using the OpenFF 2^32^ and GAFF2^55^ force fields. The other solvents (chloroform and DMSO) were parametrized for each force field in the same way as the macrocycles. It has been shown that the choice of solvent parameters can affect simulation outcomes for specific systems.^56^ Here, we have chosen to use typical default settings in order to give a fair comparison between force fields.

### Enhanced Sampling with REST2

In REST2,^35^ parts of the potential-energy function are scaled down to accelerate transitions, instead of increasing the temperature of the whole system as in standard temperature REMD.^36^ Usually, the dihedral angle terms and intramolecular nonbonded interactions of the solute are scaled in REST2 by a factor λ, while the solute–solvent interactions are scaled by √λ. Since only a small part of the system is biased, REST2 can enable stronger acceleration while retaining a reasonably high replica-exchange acceptance probability.

Typically, the λ-values are distributed either according to an exponential^57,58^ or a quadratic^46,59^ distribution. While these simple schemes work well for small systems, it has been shown in the case of proteins that the distribution of replicas might need to be adapted on a case-by-case basis.^60^ In the exponential scheme, the scaling parameters λ_*i*_ of the ensembles are chosen according to
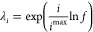
1with *i* being the ensemble index starting from 0, *i*^max^ is the highest replica index (i.e., *N* – 1), and *f* is the scaling of the highest replica (0.125 in this study).

In the quadratic scheme, λ_*i*_ values are chosen according to
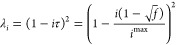
2Here, τ is the scaling factor as defined in the REAF (replica exchange with arbitrary degrees of freedom) section of the AMBER manual.^46^ It has been noted^35^ that scaling force-field parameters is conceptually similar to increasing the temperature *T* in a part of the system, corresponding to
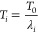
3

In this study, we compared between the two schemes and found that the quadratic scheme leads to more even replica-exchange probabilities over a large range of scaling factors. The results are shown in Figures S1–S4 in the Supporting Information. For example, the exchange rate in simulations of spiramycin in water range from 16 to 51% with the exponential scheme and from 26 to 35% with the quadratic scheme. Additionally, Figure S5 shows that including the bond-angle parameters in the scaling helps to sample the conformations of lorlatinib, a compound with a relatively short and strained macrocycle. Unless noted otherwise, the simulations in this study were performed with quadratic scaling. For lorlatinib, we additionally included the angle parameters in the REST2 scaling.

### Simulation Details

The program *gmx solvate* was used to create cubic solvent boxes with a side length of 7 nm. Water coordinates were taken from the *spc216.gro* file contained in GROMACS. Chloroform and DMSO coordinates were created from a single copy of the molecule, while adjusting the box size to create roughly the correct density (0.52 nm in each direction for chloroform and 0.58 nm × 0.45 nm × 0.45 nm for DMSO).

For all MD simulations, an integration time step of 2 fs was used while constraining bonds involving hydrogen with the LINCS^61^ algorithm. A leapfrog integrator was used, and the motion of the center of mass of the system was reset every 0.2 ps. Long-range electrostatics were treated with the PME^62^ method using a grid spacing of 0.12 nm and 4th order interpolation, while the van der Waals interactions were cut off at 1.0 nm using a shifted potential to avoid discontinuities in the potential-energy function. The temperature was kept at 300 K using the stochastic velocity rescaling (v-rescale)^63^ thermostat, and the pressure was set to 1 bar using stochastic cell rescaling (c-rescale).^64^ The isothermal compressibility parameter of the barostat was set to 4.5 × 10^–5^ bar^–1^ in water, 1.0 × 10^–4^ bar^–1^ in chloroform,^65^ and 5.5 × 10^–5^ bar^–1^ in DMSO.^66^ During production simulations, coordinates (excluding the solvent) were written every 1 ps.

For each system, 50,000 steps of steepest descent minimization were performed with a step size of 0.01 nm, stopping if the maximum force dropped below 1000 kJ mol^–1^ nm^–1^. Then, the system temperature and pressure were equilibrated using 100 ps of NVT simulation followed by 1 ns of NpT simulation.

H-REMD simulations were performed using the REST2 protocol^35,57^ implemented in the Plumed 2.8.2^67−69^ plugin for GROMACS 2022.05.^70−72^ We used 12 replicas and exchanges were attempted every 100 steps. Unless noted otherwise, scaling factors were distributed between 1 and 0.125 using the quadratic scheme. We found that REST2 performed poorly for lorlatinib due to the ring tension, and therefore employed an adapted version of the protocol for this compound, in which also the bond-angle parameters were scaled. This can be seen as a special case of generalized REST (gREST).^73^ We will refer to this protocol as bond-angle-REST2, and compare it to standard REST2 in Figure S5 in the Supporting Information.

### Analysis

All analyses were performed on the trajectories of the unbiased replica. In the literature, NOE distances between hydrogens are usually tabulated in terms of the corresponding heavy atoms. This information was translated into pairs of indices for the hydrogen atoms. Because the parametrization schemes of the different force fields lead to different atom orders, the assignment was done once per compound (using the OpenFF 2 topology) and transferred to the other topologies by aligning the molecular graphs. Prochiral hydrogens were assigned to best match the restraints, unless further information was available. NOE distances of equivalent hydrogens (e.g., in a methyl group) were averaged over the last 40 ns of each simulation as
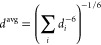
4where *d*_*i*_ is the distance between hydrogen *i* and the other atom. The violation is computed as
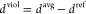
5where *d*^ref^ is the NOE upper bound from the literature data.

Clustering was performed using the average-linkage hierarchical-agglomerative algorithm implemented in the *cpptraj* program,^46,74^ using the pairwise heavy-atom RMSD as a distance metric. We created five clusters of each simulation and used the structures with the lowest distance to the centroid as representatives. Clustering is used in Figures 3 and 5 to visualize the NOE bound violations in different conformations.

## Results and Discussion

**Figure 2:**
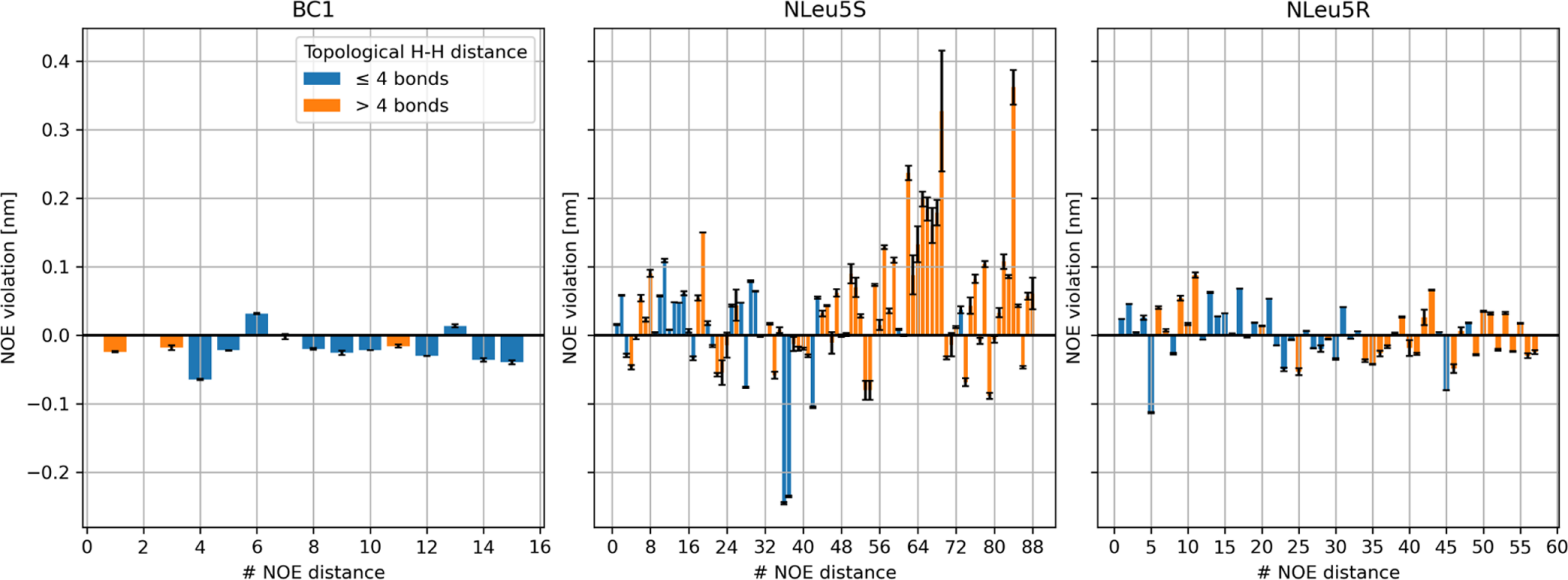
Violations of the NOE upper distance bounds for compounds BC1 (left), NLeu5S (middle), and NLeu5R (right) in chloroform for the MD ensembles generated using OpenFF 2. The bars represent the ensemble average, while black lines represent the error bars obtained by trajectory splitting. Bars are highlighted in orange if the respective hydrogen atoms are separated by more than four bonds.

For the 11 molecules shown in Figure 1, experimental NOE data in chloroform were available. To assess the statistical convergence of our simulations, we split each trajectory into five segments of equal length and performed the analysis on each segment, using the standard deviation of the five splits as an error bar. As an example for the expected range of violations, we show the detailed results for BC1, NLeu5S, and NLeu5R in chloroform using OpenFF 2 in Figure 2. The results for the other compounds are given in Figures S7–S9 in the Supporting Information.

**Figure 3:**
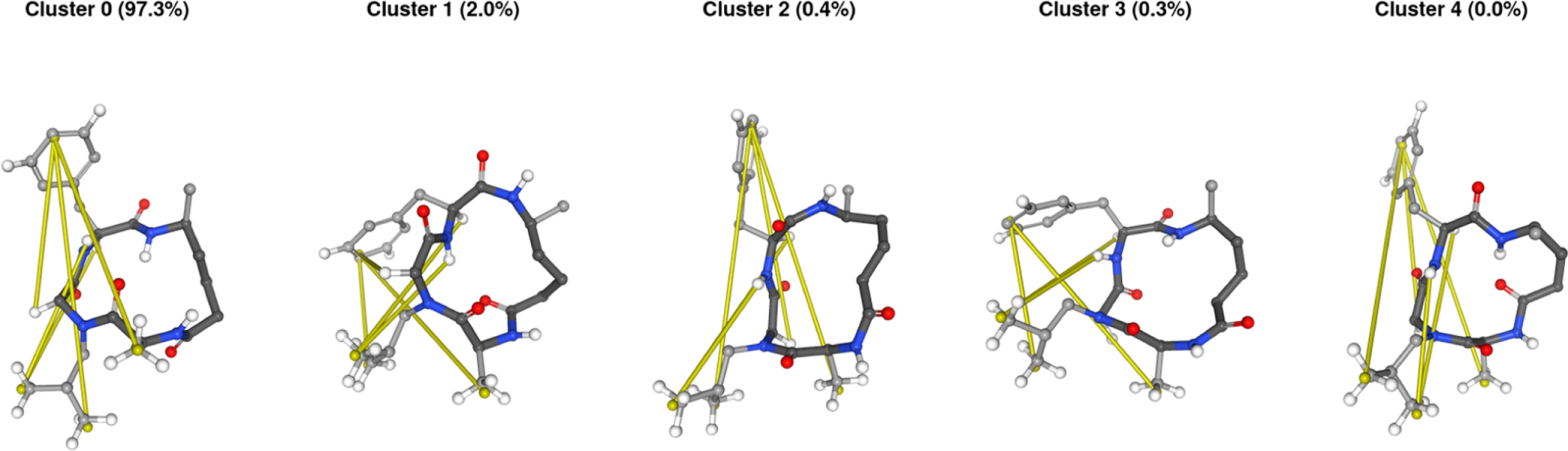
Centroid structures of the five clusters in the OpenFF 2 simulations of NLeu5S in chloroform. The distances with the five highest violations are highlighted in yellow between centers of mass of the hydrogen atoms. The corresponding hydrogen atoms as well as polar hydrogen atoms are shown as sticks.

While the BC1 ensemble matches the experimental data well, we find large violations for NLeu5S. However, the ensemble of NLeu5R, which only differs in one stereocenter, agrees well with the experiment. To investigate the high deviations of NLeu5S structurally, we performed clustering as described in the Methods section to extract five clusters. In Figure 3, the representative structures of the clusters are shown with the distances corresponding to the NOE bounds with the highest violations highlighted in yellow (note that there is a simplification for the purpose of clarity: while the NOE distances are averaged with *r*^–6^ weights for the quantitative analysis, we show the distance between centers of mass of the respective hydrogen atoms). The structure of the most populated cluster (97.3%) is similar to the one observed by Comeau et al.^41^ in simulations with the GROMOS force field,^75^ exhibiting a single intramolecular hydrogen bond between the nitrogen atom of the linker and the carbonyl of the alanine. To fulfill the experimental constraints, a significant rearrangement would be required, where the phenylalanine side chain folds toward the other side of the ring. Since none of the representative structures fulfills all NOE bounds, and some violations are shared between all five representatives, we conclude that a simple rebalancing of the probabilities would not suffice to match the simulation with experiment. Instead, very different conformations are needed, or there might be additional effects such as intermolecular interactions influencing the experiment.

### Force-Field Performance

**Figure 4:**
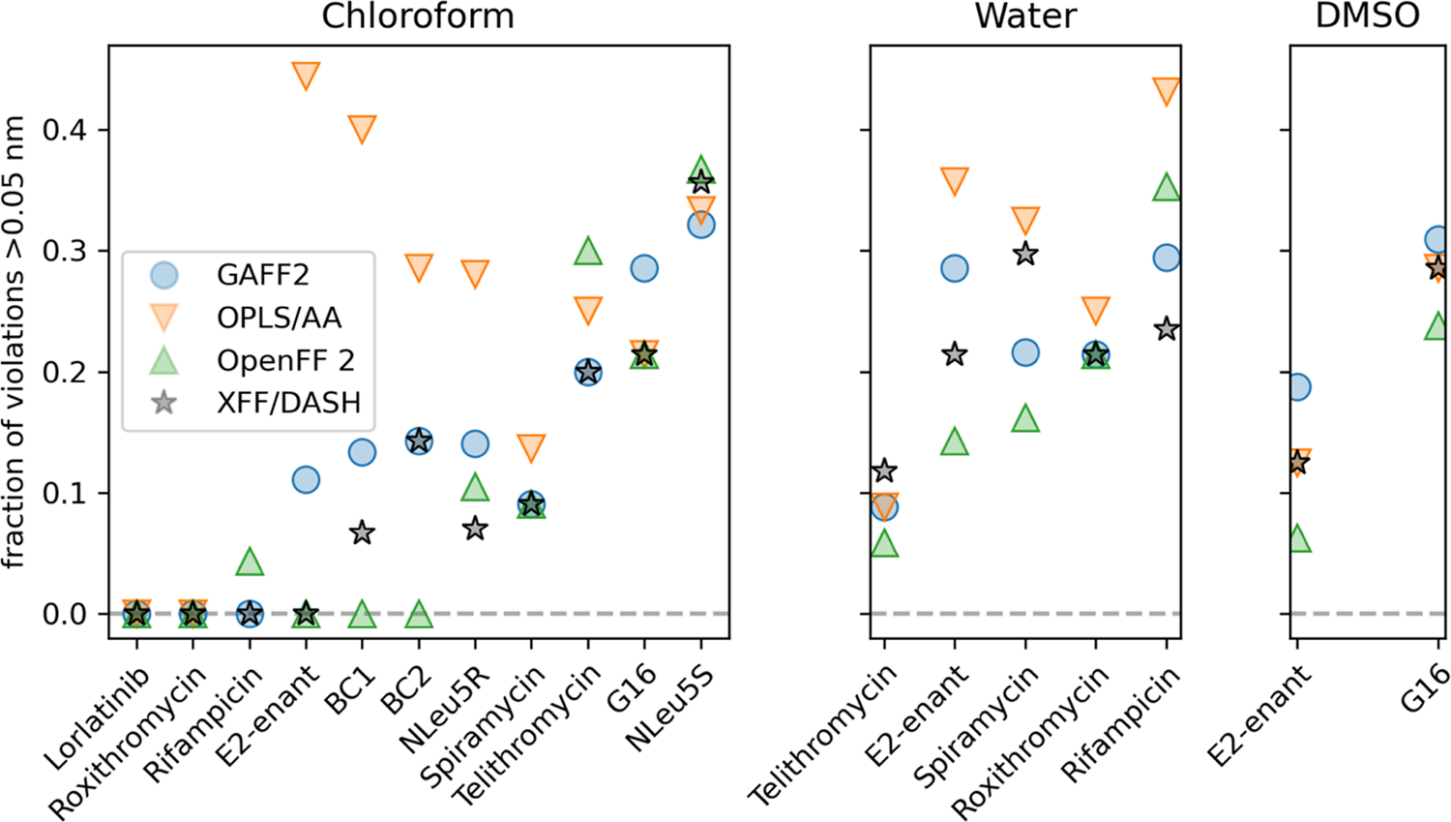
Performance of the four force fields at reproducing conformational ensembles in chloroform (left), water (middle), and DMSO (right). Each point represents the fraction of NOE violations over 0.05 nm with a given force field and compound. Note that the value for rifampicin with OPLS/AA in chloroform is missing as this molecule could not be parametrized. The compounds were sorted by the lowest respective value.

Figure 4 shows the performance of the four tested force fields across the 11 compounds as the fraction of NOE distances for which the violation is greater than 0.05 nm. In chloroform, we find that some compounds are well described by all force fields (e.g., lorlatinib and roxythromycin), while others show larger differences. For some compounds, all force fields produce significant deviations (e.g., NLeu5S). Overall, OpenFF 2 generates ensembles with the lowest number of NOE violations. With this force field, we do not find a single violation above 0.05 nm for compounds BC1, BC2, E2-enant lorlatinib, and roxithromycin (although the latter two are reproduced by all four force fields).

In addition to chloroform, experimental NOE data in water was available for compounds E2-enant, spiramycin, rifampicin, roxithromycin, and telithromycin, as shown in the middle panel of Figure 4. NOE data in DMSO was available for compounds E2-enant and G16 (right panel of Figure 4). Similar as before, we find that OpenFF 2 performs best on all compounds except rifampicin and roxithromycin, where it performs as well as XFF/DASH and GAFF 2 for the latter.

Figure S10 shows the same analysis, but splits the data into NOE bounds belonging to the macrocyclic portion of each molecule, the extracyclic portion, or a mix of both. Overall, the macrocyclic portion is reproduced better than the mixed and extracyclic ones. One possible explanation is the higher conformational restraint on the macrocyclic portion. Additionally, an incorrect conformation in the macrocycle would likely shift the relative positions of side chains, which would in turn lead to violations in the mixed portion, which might explain the higher violations.

As an overall measure of force-field performance, we computed the average fraction of violations of all simulations performed with each force field. We find overall fractions of violations of 18.8, 19.0, 20.2, and 27.1% for OpenFF 2, XFF, GAFF2, and OPLS/AA, respectively.

To test whether these differences are statistically significant, we performed a Friedman test on the combined data in Figure 4, excluding rifampicin in chloroform because of the missing data point. We find a *p*-value of 0.004 indicating that there is a statistically significant difference between force fields. We then performed pairwise Wilcoxon tests to see which differences would be significant with a *p*-value below 0.05. We find that OpenFF 2 performs significantly better than GAFF2 and OPLS/AA, and that OPLS/AA also performs worse than GAFF2 and XFF, while the differences between other pairs are not statistically significant.

### Performance across Different Solvents

**Figure 5:**
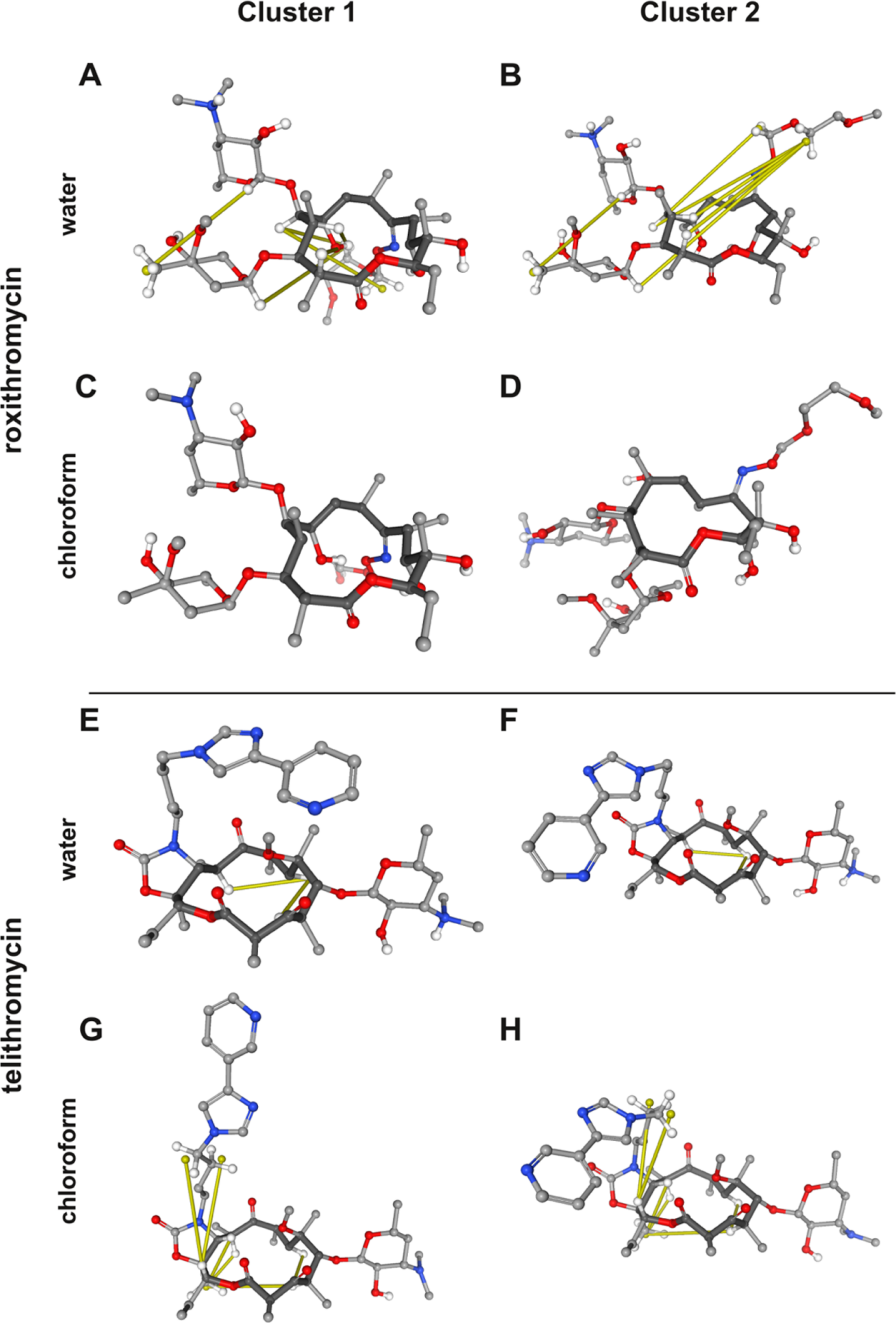
Centroid structures of the MD simulations of roxithromycin (A–D) and telithromycin (E–H) in water (A, B, E, F) and chloroform (C, D, G, H) using OpenFF 2. Violations that average more than 0.05 nm are highlighted in yellow.

Comparing the force-field performance in water with the values for the same compound in chloroform, we find contrasting results for roxithromycin and telithromycin. To rationalize these results, we performed a clustering of each trajectory (as described in the Methods section) to extract five clusters and compare between the representative structures. In Figure 5, we show the two most populated clusters of roxithromycin and telithromycin in water and chloroform. We note that these clusters represent at least 85% of the simulation time in all cases.

The most populated conformations (cluster 0) of roxithromycin in water and chloroform are very similar, and account for 99.3 and 99.9% of the simulation time, respectively. In chloroform, this is consistent with previous findings showing that roxithromycin predominantly adopts a single conformation in chloroform,^39^ and our MD data agrees well with the experimental data (Figure 5C,D). In water, however, roxithromycin has been shown to adopt multiple conformations, so the probability of the main conformer should be lower. Furthermore, the NOE upper bounds indicate that there should be additional contacts in water (Figure 5A,B).

For telithromycin, all four force fields perform well in water, with a fraction of violations over 0.05 nm of about 0.1. In chloroform, however, all force fields exhibit a fraction of violations of 0.2 or higher. The two most populated clusters are more folded in water (Figure 5E,F), whereas they are rather open in chloroform (Figure 5G,H). However, there are multiple NOE bound violations in chloroform, indicating that the conformation should also be more folded and exhibit additional intramolecular contacts.

In summary, the conformations of both roxithromycin and telithromycin are sensitive to the environment, i.e., chloroform or water, and inaccurate populations of folded and unfolded conformations seem to contribute to the deviations between the computed ensembles and the experimental NOE data.

We note that a good portion of recent force-field development focused on dihedral-angle parameters,^32,33^ and the results in Figure 4 indicate that this indeed improves the ensemble quality. In the cases of roxithromyin and telithromycin, however, strong rearrangements due to the solvent environment are not sufficiently described. This indicates that a good balance of solvation effects, and therefore intermolecular interactions, might be relevant for further force-field improvements.

### Convergence Assessment

To assess whether the REST2 protocol and simulation length was sufficient, we calculated the standard deviation from trajectory splitting for each NOE distance. The root-mean-square of the standard deviations over all NOE distances of each compound are reported in Table 2 to obtain an impression of the expected sampling uncertainties, indicating that the simulations are converged. Of course, it cannot be excluded that conformational changes may occur, which are slower than the chosen simulation length or separated by energy barriers that are too high for the chosen sampling-enhancement scheme. The latter point was illustrated by the example of lorlatinib in Figure S5 in the Supporting Information, where the correct conformer was only sampled when incorporating bond-angle terms in the REST2 protocol.

**Table 2:** Root-Mean-Square Standard Deviation of Individual NOE Distances for Each Compound in the Three Solvents, in nm

compound	chloroform	water	DMSO
BC1	0.002		
BC2	0.006		
E2-enant	0.003	0.003	0.009
G16	0.007		0.028
lorlatinib	0.0004		
rifampicin	0.010	0.017	
roxithromycin	0.0003	0.006	
spiramycin	0.025	0.012	
telithromycin	0.005	0.005	
NLeu5R	0.003		
NLeu5S	0.015		

### Performance on In-House Compounds

**Figure 6:**
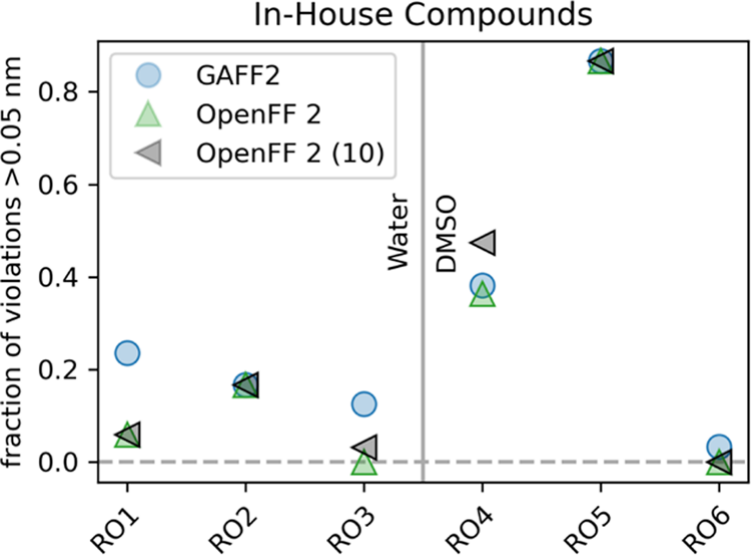
Performance of the force fields GAFF2 (blue) and OpenFF 2 (green with partial charges from one conformer, and gray with charges averaged over ten conformers) at reproducing conformational ensembles of the six in-house compounds in water (RO1–RO3) and DMSO (RO4–RO6). Each point represents the fraction of violations over 0.05 nm with a given force field and compound.

We further tested our protocol on six in-house macrocycles of different size using the OpenFF 2 and GAFF2 force fields. For three of them (RO1–RO3), NOE distances have been measured in water, while the three others were measured in DMSO (RO4–RO6). The results are summarized in Figure 6. While the ensembles in water are well predicted with both force fields, two of the three ensembles in DMSO show high numbers of NOE violations. The poor results in DMSO stem likely from the larger ring size combined with the aromatic rings within the macrocycle. From the NMR structure solution (determined using NAMFIS^76^), we find that these two molecules feature π–π interactions between aromatic rings in the backbone and side chain, which results in a major conformation that is not stable with a classical fixed-charge force field. Although fixed-charge models are inherently limited in their ability to describe complex electrostatic interactions, we note that basic π-stacking interactions can be described,^77^ so it might be possible to improve these cases by refitting the nonbonded parameters for aromatic atoms.

### Effect of the Charge Model

In the above sections, we used the default AM1-BCC routine in the OpenFF toolkit to compute charges for OpenFF 2. However, this routine only uses a single conformer to compute charges, and it has been shown previously that this can introduce bias in the simulations by differently polarizing parts of the molecule depending on their interactions in the starting conformer (which influences also the sampling of dihedral angles via the electrostatic 1,4-interactions).^78^ The documentation to OpenEye’s ELF method^79^ argues that the largest differences stem from strong intermolecular interactions.

**Figure 7:**
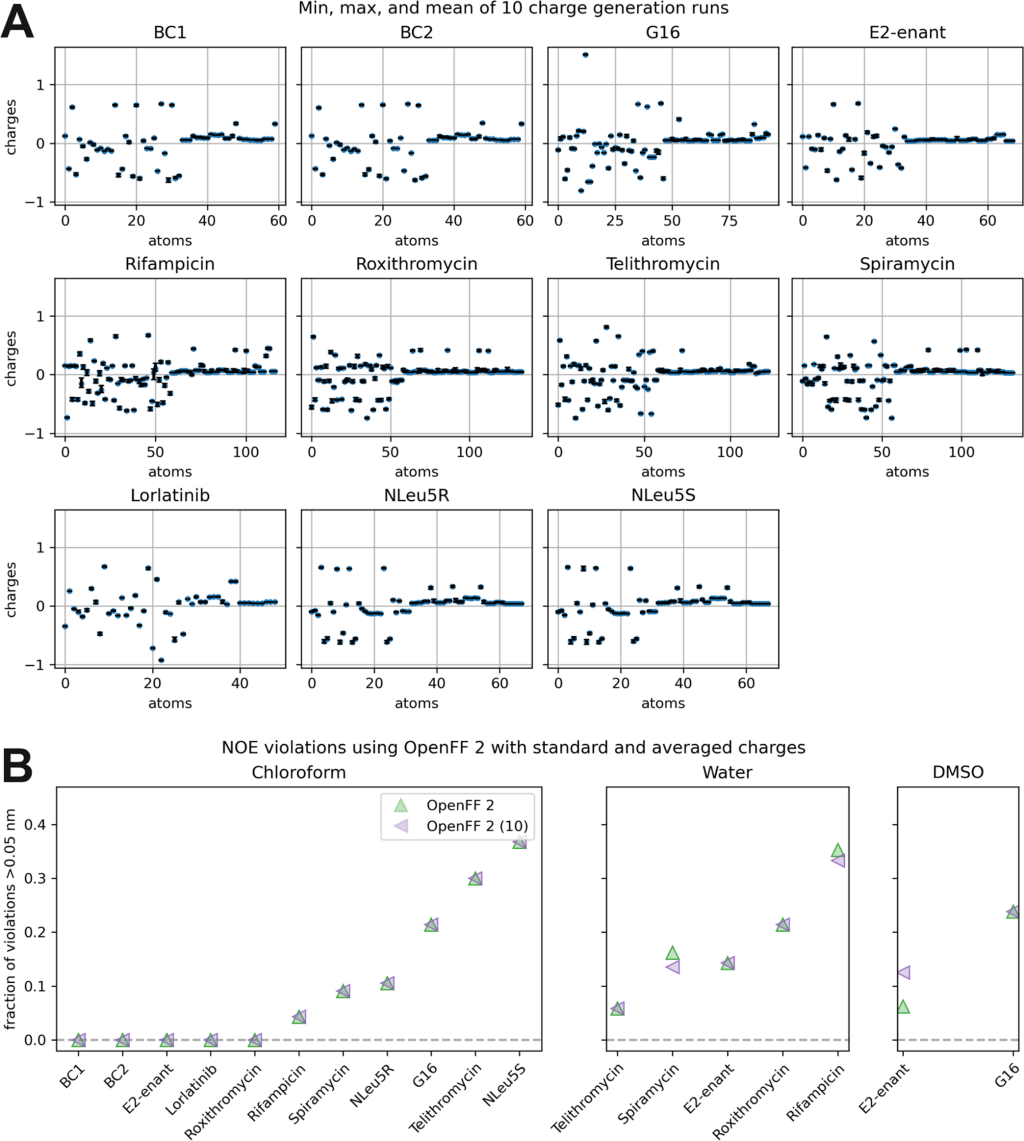
(A) Average partial charges on each atom computed from 10 different conformers (blue points), and the range from the lowest to the highest value of each atom (error bars). (B) Comparison of the NOE violations from independent simulations started with the averaged charges (purple) and those from a single conformer (green).

Therefore, we computed the average partial charge over charges obtained from ten different conformers, and repeated all simulations with OpenFF 2 in combination with these averaged charges. In Figure 7A, we show the minimum, maximum, and mean partial charges obtained from the ten conformers. The comparison of the results using OpenFF 2 force field with the two different charge sets is provided in Figure 7B. We find only very minor differences in NOE violations in all three solvents. The largest difference in partial charge is 0.18 e for the *N*-substituted aromatic carbon atom in rifampicin, and the resulting differences in NOE violations are very small. This indicates (i) that our simulations are converged, and (ii) that the variation typically seen between starting conformers has a small effect for the molecules discussed here. Nevertheless, we argue that it is better to average partial charges over multiple conformers, or to derive them from the 2D-structure as in recently published ML-based approaches^34,80,81^ to avoid occasional strongly interacting structures which might lead to nonrepresentative charges.

## Conclusions

In this work, we present a comparative study on the performance of different small-molecule force fields at reproducing conformational ensembles of semipeptidic and nonpeptidic macrocycles in solution, as judged by the comparison to experimental upper distance bounds from NOESY experiments. The performance of four different force fields (GAFF2, OPLS/AA, OpenFF 2, and XFF) was evaluated using a set of 11 publicly available and six in-house macrocycles. Overall, we find that OpenFF 2.0 performs well. However, we also observe that solvent effects are sometimes not sufficiently described, as in the example of roxithromycin and telithromycin. This implies that the solvent parameters are crucial to obtain the correct balance between relatively open and more folded (closed) conformations, and that future force-field developments should attempt to improve the balance of nonbonded and bonded parameters.

Our results suggest that modern force fields such as OpenFF and XFF (here in combination with DASH partial charges) fit experimental NOE bounds better than those obtained from older force fields such as GAFF2 or OPLS/AA. However, some compounds are still poorly described by the force fields, and we recommend that users validate their MD simulations against experimental data whenever possible.

## Data Availability

All data and source code needed to reproduce the figures, as well as input files and topologies for the MD simulations, are available on GitHub https://github.com/rinikerlab/macrocycle-ff-validation. The MD trajectories are available from the authors on request.

## Supporting Information Available

Additional data and discussions regarding the performance of the enhanced sampling protocol; comparison between the standard REST2 and bond-angle-REST2 protocols for lorlatinib; additional information regarding the NOE upper distance bounds obtained from the literature; 2D-RMSD analysis of the simulations in chloroform; individual violations of NOE upper bounds for each simulation; comparison between the force fields reproducing NOE bounds of the macrocyclic, extracyclic; and mixed portions of the molecules (PDF)
