# Supplementary material for: Validating Small-Molecule Force Fields for Macrocyclic Compounds Using NMR Data in Different Solvents
Source: J Chem Inf Model. 2024 Oct 15;64(20):7938–48. doi: 10.1021/acs.jcim.4c01120 (PMC11523072; doi:10.1021/acs.jcim.4c01120)
Supplement: Supplementary file 1 — ci4c01120_si_001.pdf [file ci4c01120_si_001.pdf]

SUPPORTING INFORMATION

Validating Small-Molecule Force Fields for Macrocyclic Compounds Using NMR Data in Different Solvents

Franz Waibl,[a] Fabio Casagrande,[b] Fabian Dey,[b] and Sereina Riniker*[a]

[a] *Department of Chemistry and Applied Biosciences, ETH Zürich, Vladimir-Prelog-Weg 2, 8093 Zürich, Switzerland. E-mail: sriniker@ethz.ch*

[b] *Roche Pharma Research and Early Development, Therapeutic Modalities, Roche Innovation Center Basel, F. Hoffmann-La Roche, 4070 Basel, Switzerland*

Contents

S1 Performance of the Enhanced Sampling Protocol **S2**

S1.1 Comparison of Quadratic and Exponential Placement of Replicas S2

S2 REST2 Protocol for Lorlatinib **S5**

S3 Correction of NOE Upper Distance Bounds **S6**

S4 2D-RMSD Analysis **S7**

S5 NOE Violation Charts for All Simulations **S8**

S6 Force-Field Performance for Macrocycle, Extracyclic, and Mixed Portions of the Compounds **S10**

S1 Performance of the Enhanced Sampling Protocol

S1.1 Comparison of Quadratic and Exponential Placement of Replicas

For several solute/solvent combinations, we compared between the quadratic and exponential sampling protocols of REST2 as described in the Methods section. We chose G16 as a representative compound in chloroform for its wide range of functional groups. Additionally, we selected E2-enant, RO2, and spiramycin in water due to their differences in polarity and the conjugated double bonds found in spiramycin, which might introduce ring strain and slow transitions. Table S1 shows a summary of the results, while further details of each simulation are provided below.

Table S1: Round-trip times and the range of individual replica-exchange probabilities observed for compounds G16, E2-enant, spiramycin, and RO2 using OpenFF 2.

Compound	Round-trip time		Exchange rates	
	exponential	quadratic	exponential	quadratic
G16 (chloroform)	1.77	1.10	0.23–0.58	0.35–0.44
E2-enant (water)	0.30	0.31	0.32–0.64	0.41–0.52
RO2 (water)	0.31	0.29	0.15–0.50	0.26–0.35
Spiramycin (water)	1.80	1.61	0.16–0.51	0.26–0.35

Using the quadratic protocol, the distribution of replica-exchange probabilities was substantially more even, with exchange probabilities of at least 0.26 in all cases. Furthermore, there was a small improvement of the round-trip time in the simulation of compound G16.

Additionally, we evaluated the timelines of events where a simulation reaches the first or the last replica after having previously visited the other of the two. Since two successive such events constitute a round-trip, we call each of these events a “half round-trip”. Figure S1 shows half round-trips in the simulations of G16 in chloroform with both protocols, while Figure S2 shows the exchange rate at the different λ-values. The round-trip time is 1.8 ns with the exponential setup, and 1.1 ns with the quadratic one. In both cases, we find that some simulations do not reach the top or bottom replica for several nanoseconds, indicating that they are intermittently trapped in a high-energy or low-energy state. However, none of the replicas remains trapped for the entire simulation length.

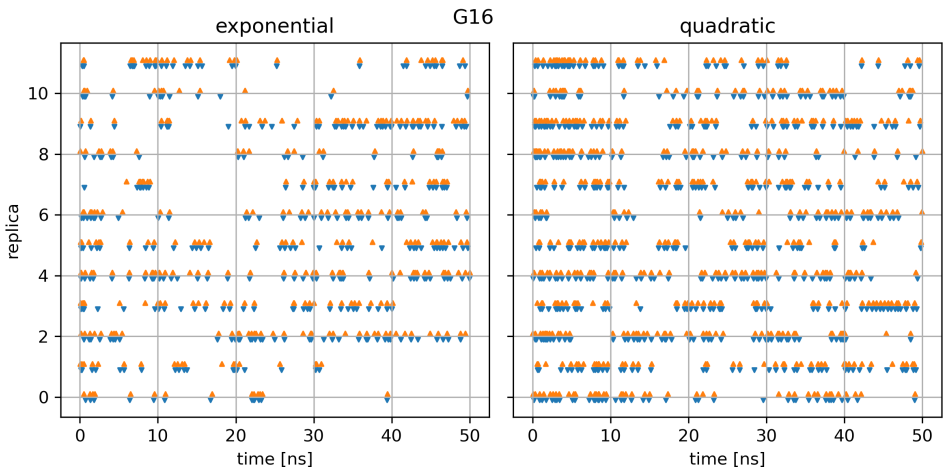

Figure S1: Occurrence of half round-trips for the compound G16 in chloroform compared between the old (exponential, left) and new (quadratic, right) protocol of distributing λ-values. Events where the lowest replica is visited are shown by blue downwards triangles, while events where the highest replica is visited are shown by orange upwards triangles.

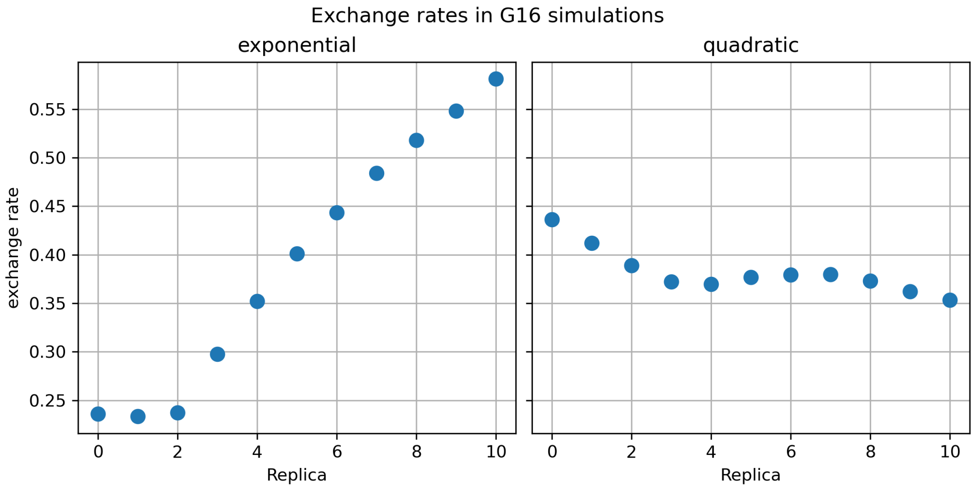

Figure S2: Success rate of replica-exchange attempts of compound G16 in chloroform compared between the exponential and quadratic protocol of distributing λ-values.

In water, we tested the quadratic and exponential protocols using three molecules with different properties: E2-enant, RO2, and spiramycin. Figure S3 shows the half round-trip events, while Figure S4 shows the success rate at different λ-values.

Compound E2-enant is relatively small and has no charged groups. As expected, both protocols perform relatively well, and we observe many round-trips. Compound RO2 is relatively large and zwitterionic. In contrast to our expectations, the charges do not negatively affect the sampling, and we observe also a large number of round-trip events for this molecule. However, the replica-exchange rate is somewhat lower than in the simulation of E2-enant. Spiramycin has a net charge of +2 in water. Additionally, it has two conjugated double bonds in the macrocycle portion, which constrain the ring flexibility. In this case,

we find significantly fewer round-trips, and sometimes a replica does not undergo any round-trips for tens of nanoseconds. This indicates that internal degrees of freedom, such as conjugated double bonds, are a larger problem than polarity in terms of sampling.

Overall, we find that the exchange rate is always more even with the quadratic protocol, while the round-trip times are slightly reduced. Therefore, we decided to employ the quadratic protocol for the rest of the study.

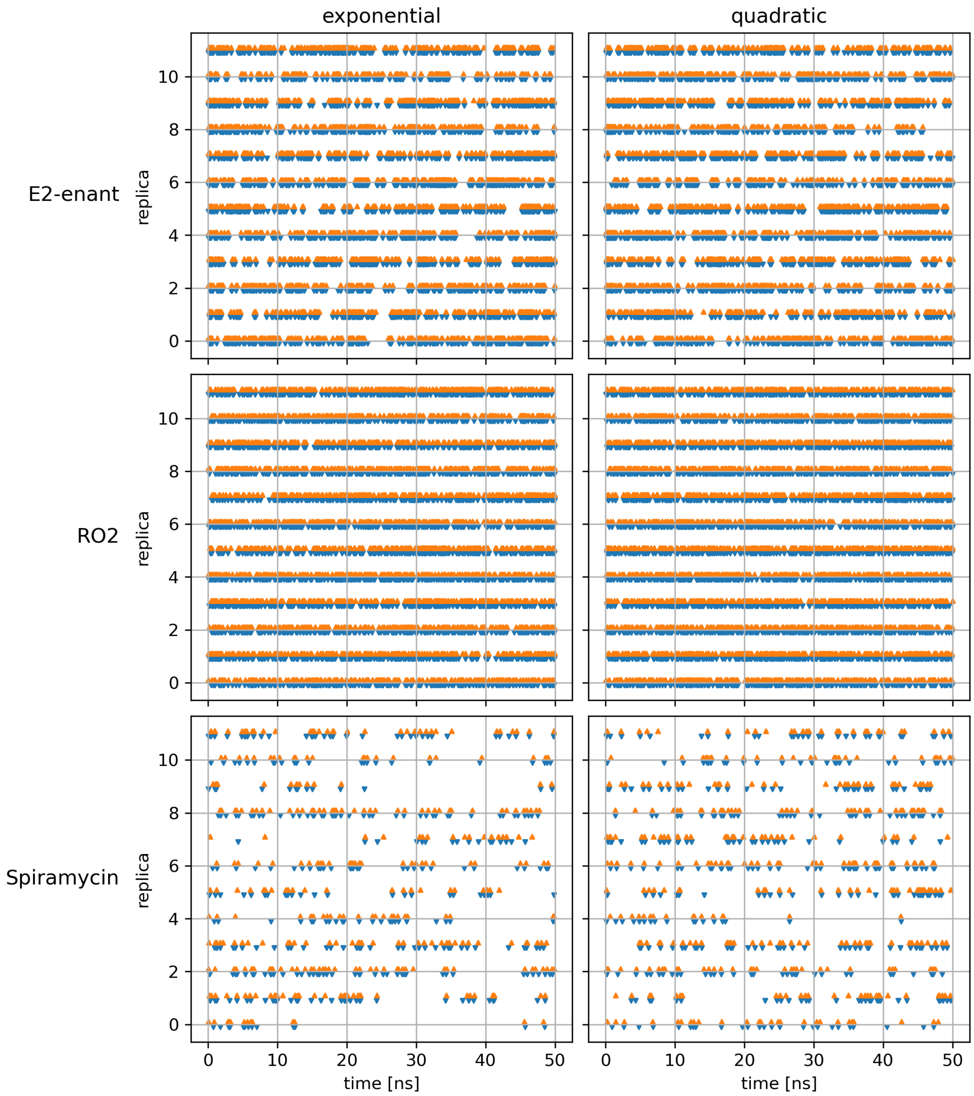

Figure S3: Occurrence of half round-trips for the compounds E2-enant, RO2, and spiramycin in water compared between the old (exponential, left) and new (quadratic, right) protocol of distributing λ-values. Events where the lowest replica is visited are shown by blue downwards triangles, while events where the highest replica is visited are shown by orange upwards triangles.

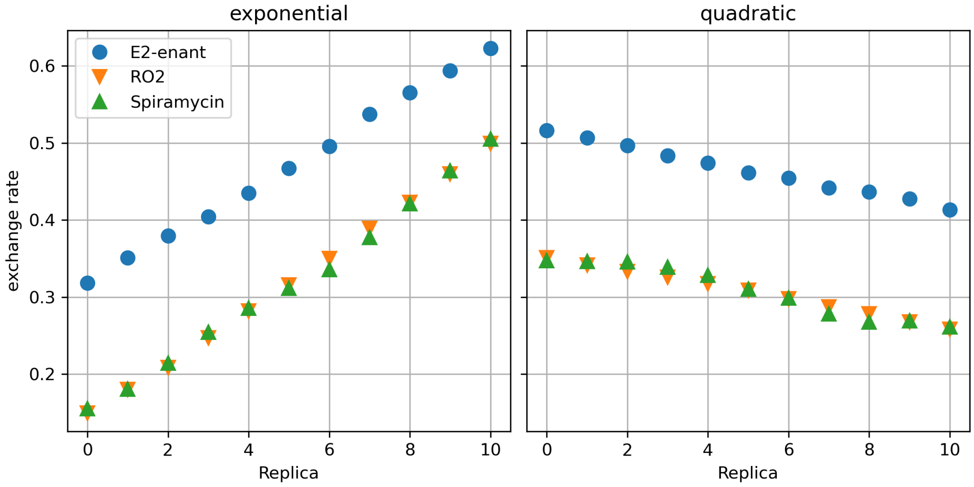

Figure S4: Success rate of replica-exchange attempts of compounds E2-enant (top), RO2 (middle), and spiramycin (bottom) in water compared between the exponential and quadratic protocol of distributing λ-values.

S2 REST2 Protocol for Lorlatinib

Since lorlatinib contains a very rigid ring structure, we found that standard REST2 cannot reliably sample transitions from the starting structure to the equilibrium conformer. With bond-angle-REST2, such transitions occurred quickly in all force fields. The improvement in NOE violations is shown in Figure S5. The bond-angle-REST2 protocol was used for lorlatinib, while the quadratic REST2 protocol was employed for the other compounds.

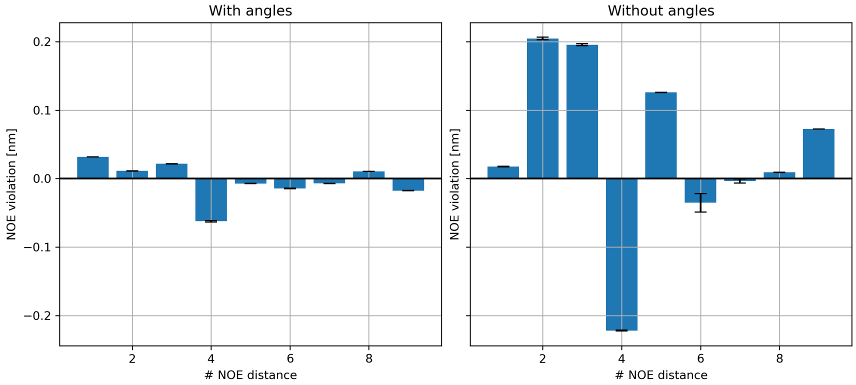

Figure S5: Violations of NOE distance upper bounds for lorlatinib in chloroform with the bond-angle-REST2 protocol (left) and the standard REST2 protocol (right).

S3 Correction of NOE Upper Distance Bounds

In three cases, we corrected the experimental NOE distance constraints after consultation with the original authors. For spiramycin in chloroform [1], we found that NOE distance no. 15 was almost impossible to fulfil. Interestingly, the same distance bound was also not fulfilled in the original NAMFIS [2] analysis. For roxithromycin in chloroform and spiramycin in water [1], we found that all NOE distance bounds were violated by similar amounts, and that some upper bounds were unreasonably low for pairs of nonpolar hydrogen atoms (e.g., 0.176 nm for NOE distance no. 15 of roxithromycin in chloroform, or 0.188 nm for NOE distance no. 8 of spiramycin in water). Again, the original NAMFIS analysis showed also violations of these bounds. Too short distances can occur when an inappropriate reference distance was chosen, which would then be propagated to all other distances.

In agreement with the original authors, we found that these bounds were inaccurate and adopted the following changes: (1) NOE distance bound no. 15 for spiramycin in chloroform was omitted. (2) All NOE distances for roxithromycin in chloroform were scaled by 25 %, and (3) all NOE distances for spiramycin in water were scaled by 28 %.

S4 2D-RMSD Analysis

Figure S6 shows 2D-RMSD plots comparing each pair of frames of the simulations in chloroform using OpenFF 2. We note that, while the entire simulations are shown here, the first 20 % of each simulation were omitted for the quantitative analyses.

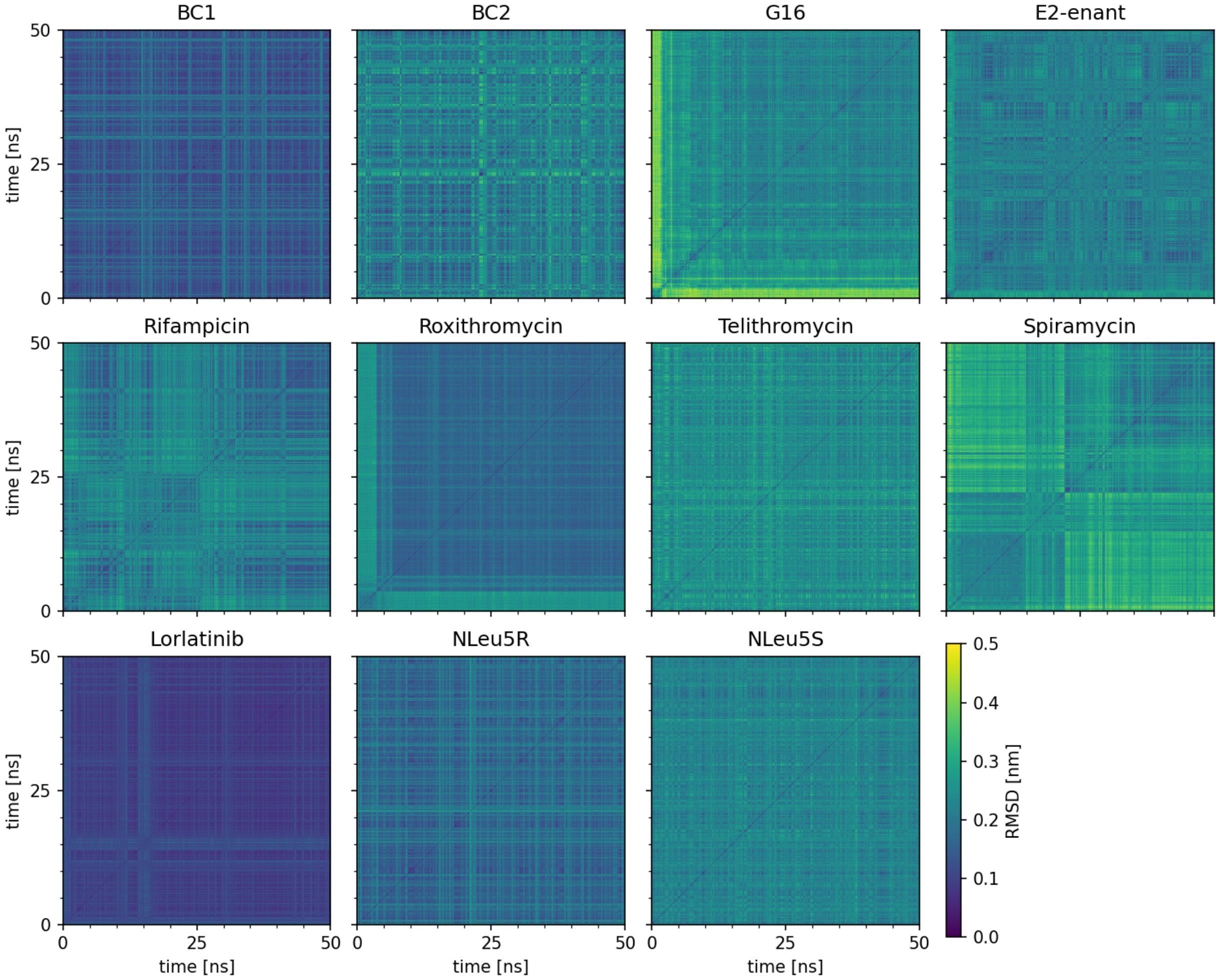

Figure S6: 2D-RMSD analysis by comparing each pair of frames of the simulations in chloroform using OpenFF 2.

S5 NOE Violation Charts for All Simulations

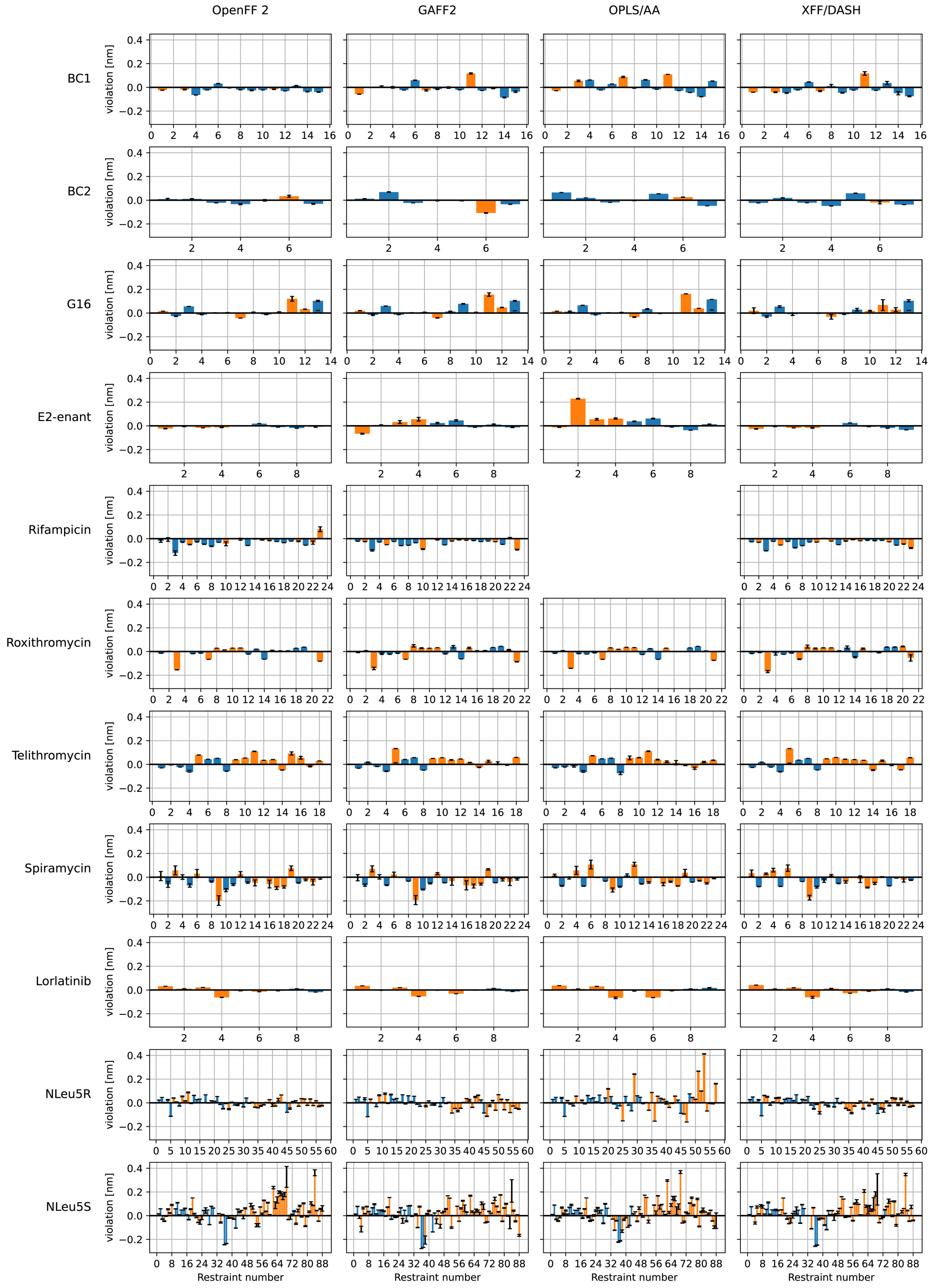

Figure S7: Bar plots of the NOE upper distance bound violations for all molecules and force fields in chloroform. The simulations of lorlatinib were performed with the bond-angle-REST2 protocol, while the others were performed using standard REST2. If the corresponding atoms are separated by more than four bonds, the bars are highlighted in orange.

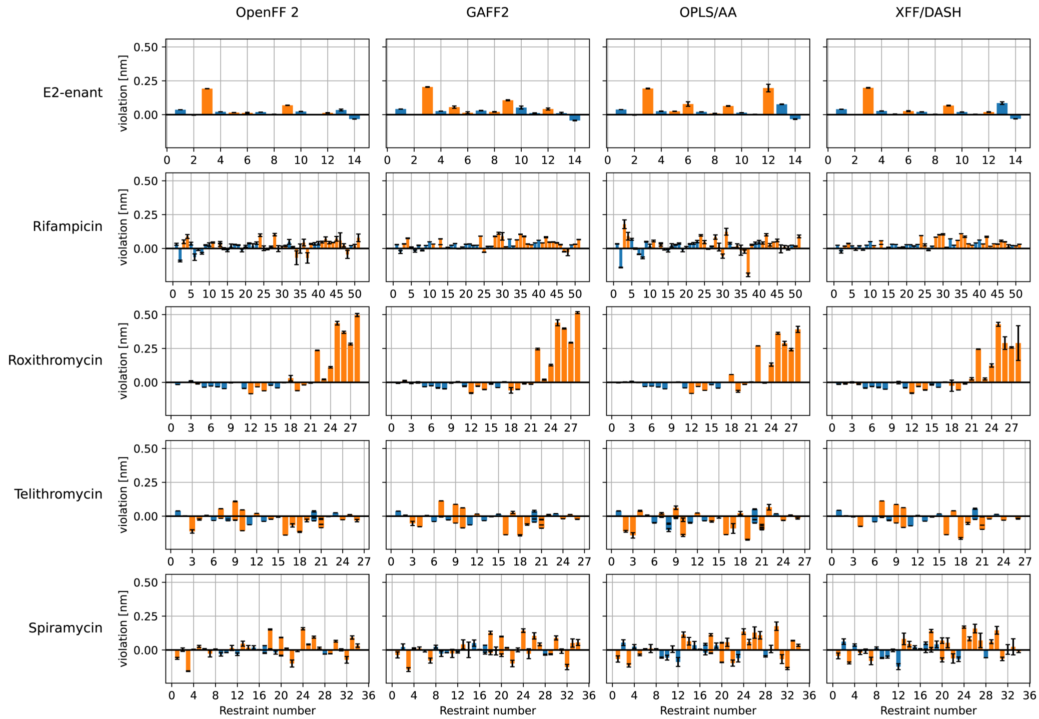

Figure S8: Bar plots of the NOE upper distance bound violations for all molecules and force fields in water. If the corresponding atoms are separated by more than four bonds, the bars are highlighted in orange.

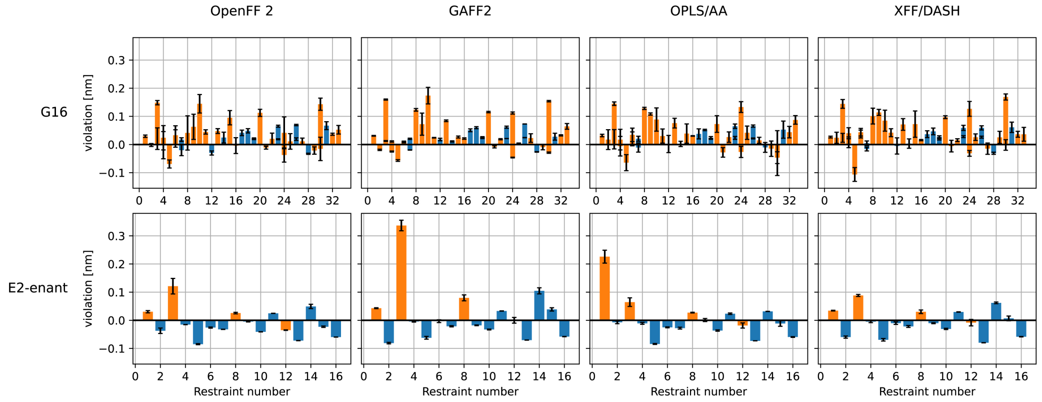

Figure S9: Bar plots of the NOE upper distance bound violations for all molecules and force fields in DMSO. If the corresponding atoms are separated by more than four bonds, the bars are highlighted in orange.

S6 Force-Field Performance for Macrocycle, Extracyclic, and Mixed Portions of the Compounds

Figure S10 shows the performance of the four force fields evaluated on different parts of the compounds. NOE distances are classified as "macrocycle" if both hydrogen atoms are connected directly to the macrocycle, "extracyclic" if they belong to the same substituent of the macrocycle, and "mixed" otherwise.

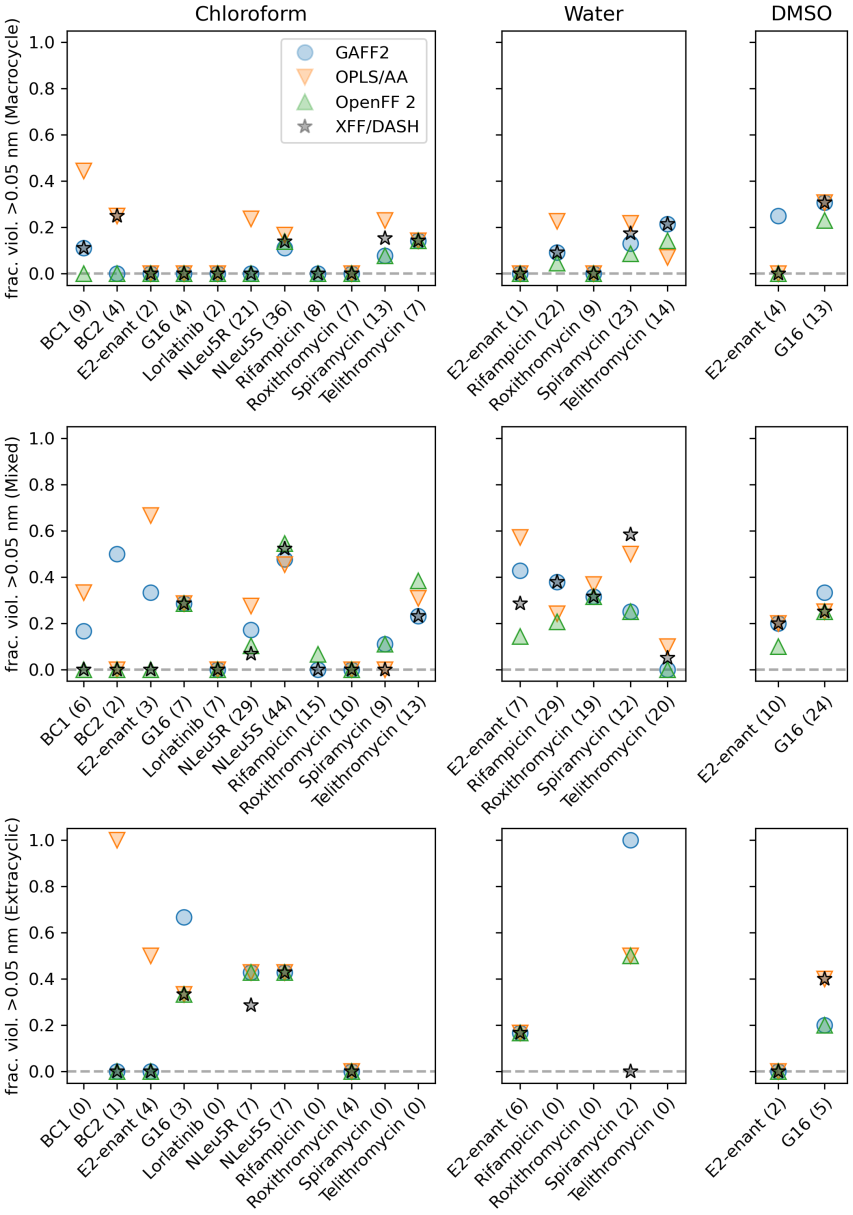

Figure S10: Performance of the four force fields on the macrocycle (top), mixed (middle), and extracyclic (bottom) portions of the eleven compounds. The x-axis labels show the number of respective NOE distances in parentheses.

References

[1] Danelius, E.; Poongavanam, V.; Peintner, S.; Wieske, L. H. E.; Erdélyi, M.; Kihlberg, J. Solution Conformations Explain the Chameleonic Behaviour of Macrocyclic Drugs. *Chem. Eur. J.* **2020**, *26*, 5231–5244.

[2] Cicero, D. O.; Barbato, G.; Bazzo, R. NMR Analysis of Molecular Flexibility in Solution: A New Method for the Study of Complex Distributions of Rapidly Exchanging Conformations. Application to a 13-Residue Peptide With an 8-Residue Loop. *J. Am. Chem. Soc.* **1995**, *117*, 1027–1033.
